# Functional rare and low frequency variants in *BLK* and *BANK1* contribute to human lupus

**DOI:** 10.1038/s41467-019-10242-9

**Published:** 2019-05-17

**Authors:** Simon H. Jiang, Vicki Athanasopoulos, Julia I. Ellyard, Aaron Chuah, Jean Cappello, Amelia Cook, Savit B. Prabhu, Jacob Cardenas, Jinghua Gu, Maurice Stanley, Jonathan A. Roco, Ilenia Papa, Mehmet Yabas, Giles D. Walters, Gaetan Burgio, Kathryn McKeon, James M. Byers, Charlotte Burrin, Anselm Enders, Lisa A. Miosge, Pablo F. Canete, Marija Jelusic, Velibor Tasic, Adrian C. Lungu, Stephen I. Alexander, Arthur R. Kitching, David A. Fulcher, Nan Shen, Todor Arsov, Paul A. Gatenby, Jeff J. Babon, Dominic F. Mallon, Carmen de Lucas Collantes, Eric A. Stone, Philip Wu, Matthew A. Field, Thomas D. Andrews, Eun Cho, Virginia Pascual, Matthew C. Cook, Carola G. Vinuesa

**Affiliations:** 1 Department of Immunology and Infectious Disease, John Curtin School of Medical Research, Acton, 2601 ACT Australia; 2 Centre for Personalised Immunology, NHMRC Centre for Research Excellence, Acton, 2601 Australia; 3 Department of Renal Medicine, The Canberra Hospital, Garran, 2601 ACT Australia; 4 Genome Informatics Laboratory, John Curtin School of Medical Research, Acton, 2601 ACT Australia; 5 Paediatric Biology Center, Translational Health Science and Technology Institute, Faridabad, 121001 Haryana India; 6 Baylor Medical Institute, Houston, 77030 Texas USA; 7 Department of Genetics and Bioengineering, Trakya University, Edirne, 22030 Turkey; 8 Department of Paediatric Rheumatology and Immunology, University of Zagreb School of Medicine, Zagreb, 10000 Croatia; 9 University Children’s Hospital, Medical School, Skopje, 1000 Macedonia; 10 Department of Pediatric Nephrology, Fundeni Clinical Institute, Bucharest, 022328 Romania; 11 Westmead Children’s Hospital, Westmead, 2145 NSW Australia; 12 Centre for Inflammatory Diseases, Department of Medicine, Monash University, Clayton, 3168 VIC Australia; 13 Department of Immunology, The Canberra Hospital, Garran, 2601 ACT Australia; 14 China Australia Centre for Personalised Immunology, Renji Hospital Shanghai, JiaoTong University Shanghai, Huangpu Qu, 200333 China; 15 Walter and Eliza Hall Institute, Parkville, 3052 VIC Australia; 16 Immunology PathWest Fiona Stanley Hospital, Murdoch, 6150 WA Australia; 17 Department of Pediatric Nephrology, Children’s University Hospital Niño Jesús, Madrid, 28009 Spain; 18 Research School of Biology and Research School of Finance, Actuarial Studies and Statistics, Acton, 2601 ACT Australia; 19 Australian Phenomics Facility, ANU, Acton, 2601 ACT Australia; 20 National Computational Infrastructure, ANU, Acton, 2601 ACT Australia

**Keywords:** Autoimmunity, Immunogenetics, Translational immunology, Systemic lupus erythematosus

## Abstract

Systemic lupus erythematosus (SLE) is the prototypic systemic autoimmune disease. It is thought that many common variant gene loci of weak effect act additively to predispose to common autoimmune diseases, while the contribution of rare variants remains unclear. Here we describe that rare coding variants in lupus-risk genes are present in most SLE patients and healthy controls. We demonstrate the functional consequences of rare and low frequency missense variants in the interacting proteins BLK and BANK1, which are present alone, or in combination, in a substantial proportion of lupus patients. The rare variants found in patients, but not those found exclusively in controls, impair suppression of IRF5 and type-I IFN in human B cell lines and increase pathogenic lymphocytes in lupus-prone mice. Thus, rare gene variants are common in SLE and likely contribute to genetic risk.

## Abstract (web summary)

Function-altering variants of immune-related genes cause rare autoimmune syndromes, whereas their contribution to common autoimmune diseases remains uncharacterized. Here the authors show that rare variants of lupus-associated genes are present in the majority of lupus patients and healthy controls, but only the variants found in lupus patients alter gene function.

## Introduction

Systemic lupus erythematosus (SLE) is a highly heterogeneous autoimmune disease in terms of both clinical phenotypes and underlying pathophysiology. Examination of disordered immune function in patients and animal models of lupus have implicated a diverse range of mechanisms contributing to disease. B-cell hyperreactivity and breaks in B-cell tolerance caused by B-cell intrinsic and extrinsic factors often involving TLR7 signaling are central to disease pathogenesis^1–4^. The most consistent final common feature in human SLE patients is overproduction of type I interferon (T1 IFN)^5,6^. Monogenic interferonopathies exemplify this contribution, providing crucial insights into how mutations affecting different genes in different patients lead to excess T1 IFN and develop similar clinical manifestations that overlap with severe SLE^7^. Concordance for lupus between monozygotic twins is approximately 50%, indicating that even in sporadic disease there is a substantial genetic contribution to aetiology^8^. While familial aggregation is well recognised, inheritance follows complex non-Mendelian patterns^9^. Variants identified by genome-wide association studies (GWAS) in genes such as *STAT4*, *IRAK1*^10^, and *LYN* are plausible by their consistency with prior observations, rather than empirical evidence of pathogenicity^11^. The prevalent hypothesis to explain the observation of common allelic frequency and modest effect size is that it is the cumulative or epistatic effect of multiple GWAS single-nucleotide polymorphisms (SNPs) in addition to environmental factors that result in predisposition to SLE^12–16^. Lupus is often a devastating disease in women of childbearing age, which might argue against the aggregate contribution of common genetic variants. Another hypothesis is that in some cases SLE arises from rare genetic variants with strong effects. Rare (MAF < 0.005) and low frequency (MAF < 0.02) variants are known to contribute to complex hereditary traits^15^ and can explain sporadic disease in the case of de novo mutations^15–17^. These disease-predisposing rare variants can be in linkage disequilibrium with GWAS-identified SNPs due to co-inheritance in shared haploblocks^18^ and may contribute to the missing heritability in common autoimmune diseases.

In this study we examine the role of rare and low-frequency variants in systemic autoimmunity. We identified several rare variants in *BLK* and *BANK1* in healthy controls and patients with SLE. *BLK* variants found in SLE patients impaired the kinase activity of BLK. We demonstrate that BLK is capable of repressing IRF5-mediated interferon-β expression (IFNβ), and that loss of BLK kinase activity caused by the rare variants enhanced interferon-β production. In contrast rare variants in *BLK* found exclusively in healthy controls did not substantially impair interferon-β repression. As expected, SLE patients with rare *BLK* variants also had increased expression of interferon signature genes compared to healthy controls. Mice bearing a *Blk* variant orthologous to one found in an SLE patient have exacerbated accumulation of pathogenic lymphoid cells when crossed to lupus-prone mice. We observe that low-frequency mutations in *BLK*’s epistatic partner *BANK1*, impair BANK1′s ability to sequester IRF5 from the nucleus. Together these data identify a novel function for BANK1 and BLK in repression of type I IFN and demonstrate how rare variants in SLE risk genes increase type 1 interferon activity which is central to development of autoimmunity.

## Results

### SLE patients have rare coding variants in SLE-risk genes

To investigate the prevalence of rare variants in lupus-risk genes we selected an initial 69 SLE probands (SLE1), a replication cohort of 64 SLE probands (SLE2) and 97 healthy elderly individuals without a history of chronic disease. The SLE cohort comprised pediatric-onset (14%), adolescent-onset (26%), and adult-onset (55%) SLE and four patients whose age of disease onset was unknown. The genetically determined ethnicity of the two cohorts was predominantly European with 79.7% of SLE patients and 100% of healthy controls of European ethnicity (Supplementary Data 1). Healthy controls underwent whole-genome sequencing (WGS) as did 12.7% of SLE patients, and the remainder whole-exome sequencing (WES). We identified rare (minor allele frequencies (MAF) below 0.005 specific to the individual’s ethnicity) missense and splice site (bases up to +/−6 from the exon border) variants.

**Fig. 1:**
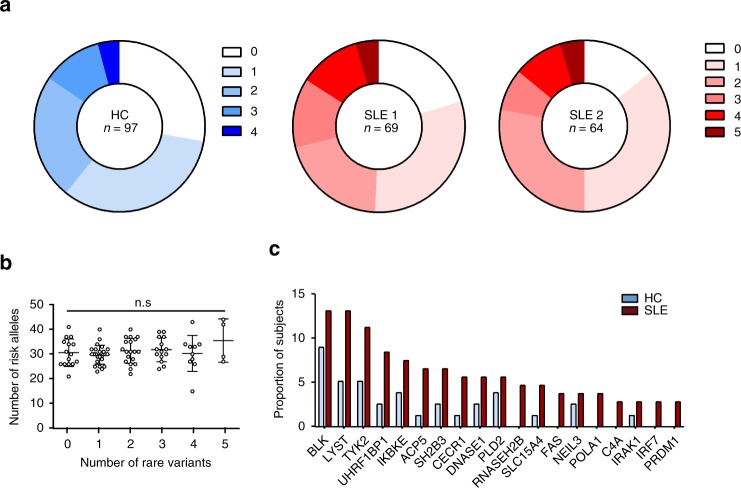
Large burden of rare nonsynonymous SNV in SLE-related genes in SLE patients. **a** Pie chart demonstrating burden of variants in two SLE cohorts, SLE1 and SLE2, and 97 elderly healthy controls (HC). The numbering 0–4 indicates the number of rare variants found in a list of 76 GWAS SLE genes. **b** Number of SLE GWAS risk alleles in individual SLE patients with indicated number of rare variants (mean and SD, Kruskal–Wallis test). **c** The most frequently mutated genes in SLE cohort of 113 probands, shown as the number of total SLE patients and healthy controls (proportion of subjects) with variants in each individual gene

We generated a list of all identified lupus-associated genes including all monogenic causes of SLE^10,19,20^, known causes of interferonopathies^21^ which share many features of SLE, and all GWAS SLE loci for which there are reported human expression quantitative trait loci, or occur in a coding region, or for which there is evidence of association with lupus from mouse studies^10,19,22^. This resulted in a list of 76 SLE genes (Supplementary Data 2). Totally, 82% of SLE patients carried rare nonsynonymous variants in SLE-associated genes as did 72% of healthy controls. In all, 48% of all patients had 1 or 2 rare variants, 30% had 3–5, and 3% had >5 rare variants. Variants found in both SLE patients and healthy controls were predominantly missense mutations. There was no difference in the distribution of the type of nonsynonymous variants (missense, nonsense, and splice site) between patients and healthy controls (Supplementary Fig. 1). SLE patients harboured on average two rare variants in lupus-gene loci (Fig. 1a). Comparison of rare variant and Immunochip SLE-associated common variants in the 76 genes did not demonstrate a correlation in the number of rare variants and common risk alleles (Fig. 1b). These findings demonstrate that rare gene coding variants in SLE-associated genes are found in most SLE patients.

### Variants in *BLK* and *BANK1* segregate with autoimmune disease

We next asked whether the rare variants observed in patients with SLE confer functional defects. For this we asked which lupus-associated genes harboured the rare variants in our cohort. Of the 76 genes, there were 20 genes with variants in ≥3 SLE patients (2.3%) and a prevalence ≥1.5 times that seen in controls (Fig. 1c, Table S2). *BLK*, *LYST*, *TYK2*, *UHRF1BP1*, and *IKBKE* were the genes most frequently containing rare variants in SLE patients. To test the contribution of rare variants to disease, we next turned to complementary functional studies.

**Fig. 2:**
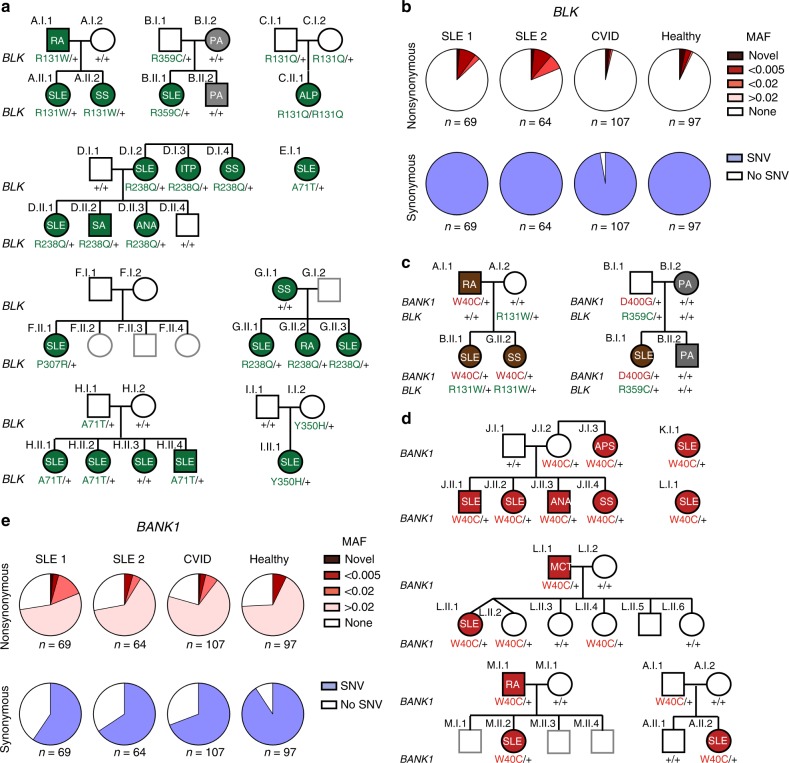
SNVs in *BANK1* and *BLK* associate with SLE. **a** Pedigrees of multiple families with low-frequency, rare, and novel SNV (single-nucleotide variant) in *BLK* associate with autoimmunity. Individuals with green shading show symptoms of autoimmunity. (ITP idiopathic thrombocytopenic purpura, ALP autoimmune lymphoproliferative syndrome, RA rheumatoid arthritis, SS Sjogren’s Syndrome, PA psoriatic arthritis, SLE  = systemic lupus erythematosus, ANA antinuclear antibodies, SA seronegative arthropathy). The amino acid position and change within the BLK protein, from the more common or “wild type”, is shown below each individual (+ = “wild type”). Individual families are indicated by (**a**–**i**). **b** Frequencies of synonymous and nonsynonymous SNVs in *BLK* in original (SLE1) and replication SLE cohorts (SLE2), common variable immunodeficiency (CVID), and healthy control cohorts. SNV single-nucleotide variant, MAF minor allele frequency. **c** The pedigrees of Family A and B identify combined variants in *BLK* and *BANK1* in SLE patients. **d** Pedigrees of families (**a**, **j**–**m**) with autoimmunity and the *BANK1*^*W40C*^ variant. MCT mixed connective tissue disease, APS antiphospholipid syndrome). **e** Frequencies of synonymous and nonsynonymous SNV in *BANK1* in SLE1, SLE2, CVID, and healthy control cohorts

We started by looking at the SNV in *BLK* given the high frequency of rare variants in this gene. *BLK* was also chosen because multiple common variants tagging *BLK* (rs2248932, MAF: 0.49; rs2736340, MAF: 038; and rs13277113, MAF: 0.36) have been identified as SLE-predisposing by GWAS (OR = 1.39 [95% CI: 1.28–1.51])^14,23,24^ and a large scale Immunochip study^25^. Also, *BLK* in humans is expressed in B cells and plasmacytoid dendritic cells^26^, two cell types considered to be important for SLE development. The *BLK* GWAS variants reported to date are noncoding and suggested to overlap with regulatory elements leading to reduced *BLK* gene expression^27^. In the initial SLE cohort we identified 6 rare or novel SNV in *BLK* (*BLK*^*R131W*^ rs73663163, *BLK*^*R131Q*^ rs144615291, *BLK*^*R238Q*^ rs141865425, *BLK*^*P307R*^ novel, *BLK*^*Y350H*^ rs758750492, and *BLK*^*R359C*^ rs146505280, and *BLK*^*R359C*^ rs146505280) in 14 patients and an additional low-frequency variant *BLK*^A71T^ (rs55758736, MAF = 0.012) in 5 patients (Fig. 2a and Table 1). Thus, 10.1% of our original SLE patients (SLE1, *n* = 7 of 69) (Fig. 2a) and 10.9% of our replication cohort (SLE2, *n* = 7 of 64) had a rare or novel missense SNV in *BLK*. Rare or novel missense SNVs in *BLK* were found at significantly lower frequencies in a common variable immunodeficiency/complex immunodeficiency (CVID/CID) cohort (*n* = 3 of 107, 2.8%, *p* ⩽ 0.02) but at comparable frequency in healthy controls (*n* = 7 of 97, 7.2%, *p* = 0.5). Synonymous SNV of any allelic frequency in *BLK* were found at equivalent rates in all three cohorts (Fig. 2b).

**Table 1:** Comparison of *BANK1* and *BLK* variants found in SLE patients

	MAF^a^	Type	AA change	CADD	HC with allele (*n*)	SLE with allele (*n*)
*BLK*						
GWAS SNPs						
rs2248932	0.61	Intron	N/A	N/A	–	–
rs13277113	0.25	Intron	N/A	N/A	–	–
rs2736340	0.25	Intron	N/A	N/A	–	–
NRLF SNPs						
rs55758736	0.01	Missense	A71T	5.86	1	7
rs73663163	0.0005	Missense	R131W	14.93	0	1
rs144615291	0.00004	Missense	R131Q	16.05	0	1 (*n* = 1 hom)
rs141865425	0.003	Missense	R238Q	22.2	2	3
N/A	Novel	Missense	P307R	21.8	0	1
rs77401687	0.002	Missense	K325T	18.18	0	1
rs758750492	0.000004	Missense	Y350H	15.7	0	2
rs146505280	0.0008	Missense	R359C	20.1	1	1
N/A	Novel	Missense	R433Q	17.35	0	1
rs202162624	0.002	Missense	R450H	18.23	0	2
*BANK1*						
GWAS SNPs						
rs10516487	0.26	Missense	R61H	13.9	56 (*n* = 26 hom)	59 (*n* = 9 hom)
NRLF SNPs						
rs35978636	0.01	Missense	W40C	29.8	6	10
rs201960198	0.0001	Missense	D400G	27.4	0	1

^a^Minor allelic frequency by gnomAD

*GWAS*  genome wide association study, *NRLF*  novel, rare and low-frequency, *Hom* homozygous, *HC* healthy control, *SLE* systemic lupus erythematosus

All relatives with systemic autoimmunity in the pedigrees of probands with *BLK* rare alleles also carried the *BLK* variants, however, 25% of individuals carrying the variants did not have disease. While there are multiple factors shown to contribute to incomplete gene penetrance (gender, environmental influences, epigenetic changes, combined effects of risk and protective common alleles, etc.), we considered the possibility of additional SLE rare variants acting in epistasis with *BLK* being present in subjects with disease. In search for variants in BLK- interacting proteins, we identified 2 families in which there was co-segregation in patients with autoimmune disease of a rare *BLK* SNV with a rare and a low-frequency mutation in *B-Cell Scaffold Protein With Ankyrin Repeats 1* (*BANK1*) (*BANK1*^*D400G*^ rs201960198, ExAC MAF < 0.00007 and *BANK1*^*W40C*^ rs35978636, ExAC MAF:0.0103), respectively). *BANK1* expression is also restricted to B cells and plasmacytoid dendritic cells^28,29^, is associated with SLE by GWAS^12^ and a large scale Immunochip study^25^, and has been reported to function in epistasis with *BLK*^30^ (Fig. 2c).

Interestingly, we noted that the GWAS-associated SNP (4:101829919 G/A; *BANK1*^*R61H*^, MAF 0.25, odds ratio (OR) = 1.4 [95% confidence interval (CI): 1.3–1.5], *p* = 3.74 × 10^−^^10^), tags a common *BANK1* haplotype^12,14^ and is likely in linkage disequilibrium with the low-frequency *BANK1*^*W40C*^ in our SLE cohort (Supplementary Fig. 2); both SNVs occur within exon 2. We hypothesised that, like the *BANK1*^*R61H*^ GWAS SNP, *BANK*^*W40C*^ may be observed at high frequency in SLE patients. Indeed, when we searched for this variant in a third cohort comprised of 150 SLE patients (for which the DNA yield and quality was insufficient for WES, but adequate for a targeted polymerase chain reaction (PCR)-amplifluor assay), we identified the *BANK1*^*W40C*^ SNV in 9 SLE probands (6%) and in 3 out of 222 unaffected controls (*n* = 3 of 222, 1.3%) resulting in an OR of 4.7 (95% CI: 1.1–27.1, *p* = 0.017) (Fig. 2d). No further low-frequency or novel *BANK1* variants were found in the SLE cohort (Fig. 2e). Together these data demonstrate that a substantial fraction of SLE patients have one of multiple rare- or low-frequency SNVs in *BANK1* and/or *BLK*.

### *BLK* SNVs impair phosphorylation of BANK1 and IRF5

**Fig. 3:**
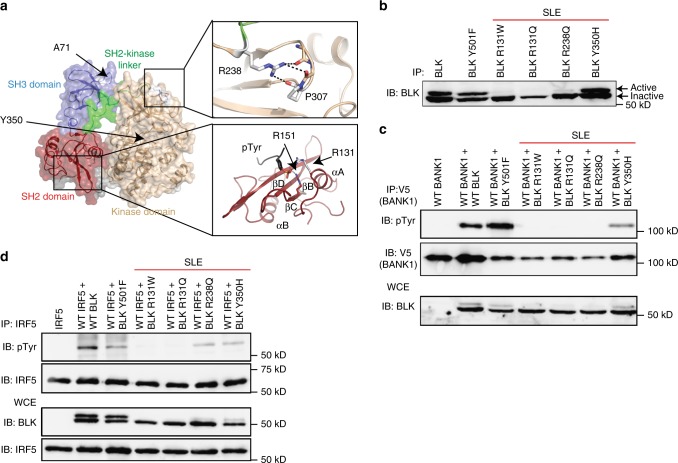
Rare and novel *BLK* variants are deleterious and impair kinase activity. **a** Modelled structure of the wild type BLK SH2 domain (PDB 1BLK) shown with a bound phosphotyrosine (pTyr) ligand (modelled from PDB 1 × 27). Arginine 131 (shown in white stick representation) is located on Helix ɑA (ɑA) and is known to co-ordinate phosphorylated tyrosine residues in a number of SH2 domain:ligand structures alongside the conserved R151 at helix ɑB (R151 in BLK, red stick representation). The other residues found to be altered in patients namely Y350 and R238, which interacts with P307, are also shown. Predicted polar contacts are shown as dotted lines. Beta-sheets are shown as ribbons with arrows and labeled βB-βC. Src Homolgy 3, SH2; Src Homology 3, SH3. **b** Anti-phosphotyrosine blotting of overexpressed and immunoprecipitated BLK^WT^, BLK^Y501F^, and BLK^R131W^ in HEK293T cells. The lower molecular weight band corresponds to the inactive form of BLK and the upper band to the active form. IP immunoprecipitation, IB immunoblot, WCE whole-cytoplasmic extract, SLE systemic lupus erythematosus. **c** Antiphosphotyrosine blotting of BANK1 after co-transfection of HEK293T cells with *BANK1* and *BLK* expression vectors and pulldown of BANK1. pTyr phosphotyrosine, **d** Blotting of IRF5 after co-transfection of IRF5 and BLK variants in HEK293T cells indicates impaired phosphorylation of IRF5 by identified BLK variants. Blots in **b**–**d** are representative of at least three experiments

We next examined the effect of the *BLK* and *BANK1* SNVs on protein function. We noted that many of the *BLK* SNVs identified are in functionally important regions of the protein. The arginine residue at position 238, mutated in families D and G, is strictly conserved in all Src-family kinases. In the resolved protein crystal structures this residue is seen to orient the SH2-kinase linker with the N-lobe of the kinase domain via an interaction with P307^31^, which is mutated in family N (Fig. 3a). Mutation of the analogous R238 residue in Src has been shown to inhibit its catalytic activity^31^. In addition, the arginine residue at position 131, mutated in families A and C, is conserved in many SH2 domains and assists in the coordination of bound phosphotyrosines. The tyrosine at 350, mutated in family J, resides within the kinase domain and we hypothesized would restrict BLK catalytic activity. *BLK*^*A71T*^ is known to reduce protein stability and thus minimize available protein^32^. Therefore, we postulated that the identified SNVs would alter kinase activity of BLK by impairing availability of protein or protein function. *BLK* expressed in heterologous cell lines translates a higher molecular weight active band due to relatively higher phosphorylation, and a lower molecular weight inactive band^33^. As expected, upon transfection of *BLK*^*R131W*^, *BLK*^*R131Q*^ and *BLK*^*R238Q*^ into HEK293T cells almost no active bands were seen, compared with visible active bands in cells transfected with wild type *BLK*, *BLK*^*Y305H*^ and a constitutively active *BLK*^*Y501F*^ (Fig. 3b). This suggests that some of the *BLK* mutations impair the protein’s ability to acquire an active conformation. As BLK is known to phosphorylate BANK1 and SNVs were identified in both genes in the three patients, we tested whether the identified BLK variants had impaired ability to phosphorylate BANK1. Indeed, the four rare *BLK* variants tested had impaired phosphorylation of BANK1 (Fig. 3c). Collectively, this demonstrates that rare variants in BLK identified in a large proportion of SLE patients have significantly impaired kinase function.

### *BLK* SNV impair repression of IRF5-mediated T1 IFN expression

We then explored the contribution of rare alleles of *BLK* to the pathogenesis of SLE. Since BLK activates PLCG2 and induces Ca^2+^ flux upon B-cell receptor (BCR) signaling^34^, we tested whether the variant may impair Ca^2+^ flux in a human B-cell line (Ramos) in which we introduced the *BLK*^*R131W*^ variant using CRISPR-Cas9 editing. No difference in response to BCR-mediated Ca^2+^ flux was observed in *BLK*^*R131W/R131W*^ cells compared with WT cells, excluding this as a potential mechanism (Supplementary Fig. 3).

BLK is a member of the SrcB kinase subfamily, which includes LYN. LYN and BLK are both activated by CD79-mediated phosphorylation upon BCR cross-linking^35^, and both phosphorylate BANK1 after BCR stimulation^34^. Common alleles of *LYN*, like *BLK*, have been implicated in SLE by GWAS^13^ and *Lyn* deficiency induces a lupus-like phenotype in mice^36^. Besides regulating calcium flux^36^, *Lyn* deficiency also promotes autoimmunity through impaired regulation of IRF5-mediated T1 IFN production and activity^11^. We thus hypothesized that BLK may share IRF5 as a substrate with LYN and the hypomorphic *BLK* variants may impair IRF5 regulation. Indeed, upon co-expression of *BLK* and *IRF5* in HEK293T cells, wild-type BLK phosphorylated IRF5, whereas the identified rare BLK variants had diminished or no ability to phosphorylate IRF5 (Fig. 3d).

**Fig. 4:**
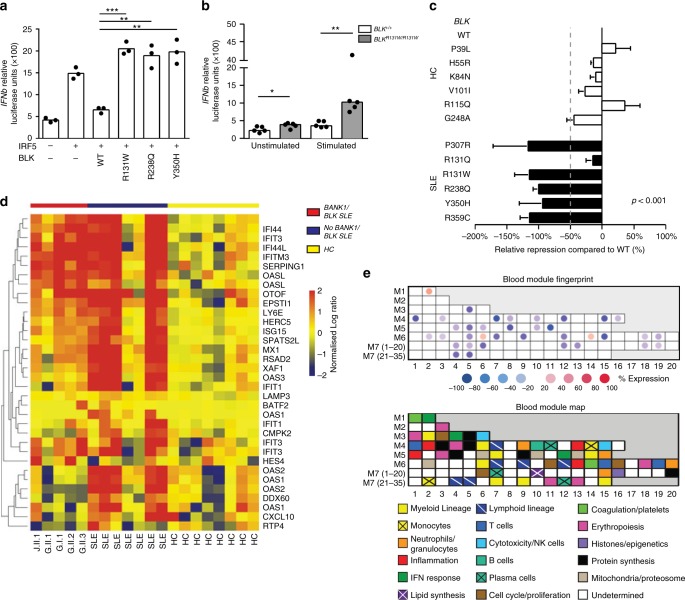
*BLK* variants fail to repress type 1 IFN expression. **a** IFNβ luciferase activity 24 h after co-transfection of HEK293T cells with IRF5, MyD88, and indicated BLK variants. Each data point represents the relative luminometer unit (RLU) relative to a cotransfected control plasmid expressing renilla luciferase, and is the average of three technical replicates. (*p < 0.05, **p < 0.01, ***p < 0.001 Student *t* test). **b** IFNβ luciferase expression in CRISPR-Cas9 edited Ramos cells homozygous for the R131W variant, 24 h after stimulation with resiquimod (R848). Each data point represents the relative luminometer unit (RLU) relative to a cotransfected control plasmid expressing renilla luciferase, and is the average of five technical replicates. (*p < 0.05, **p < 0.01, Mann–Whitney *U*). **c** IFNβ luciferase activity relative to WT BLK 24 h after co-transfection of HEK293T cells with IRF5, MyD88, and indicated BLK variants found in healthy controls (HC) or SLE patients (SLE). Data points for each variant were normalized to the repression levels of WT BLK. The dotted line indicates 50% of repression by WT BLK (mean and SD). **a**–**c** representative of at least three experimental replicates (*p* < 0.0001, two-tailed ANOVA). **d**. Heat map of 34 hierarchically clustered genes within IFN module M1.2. The data are normalized to healthy controls by subtracting out the mean of the healthy controls across all samples. The samples are ordered by condition, illustrating IFN upregulation in both BANK/BLK and non-BLK patients relative to the healthy controls. **e** For BLK samples, the map shows the percentage of genes within each module (minimum 10%) that are significant (False discovery rate (FDR) ≤ 0.05) and overexpressed (red) or underexpressed (blue) relative to healthy controls. As an example, a module in which 50% of the genes are significantly upregulated and 25% are significantly downregulated would appear on the map as 50–25% = 25% upregulated and would have a light red color

We next tested whether, as shown for LYN, BLK plays an active role in the repression of IRF5 and T1 IFN activity. Expression of the *BLK* variants with an *IFNb* dual luciferase reporter demonstrated that all tested BLK variants (*BLK*^*R131W*^, *BLK*^*R238Q*^, and *BLK*^*Y350H*^) were unable to repress IRF5-mediated *IFNb* activity compared to wild-type BLK (Fig. 4a) in a dose-dependent manner (Supplementary Fig. 4). In addition, using a CRISPR/Cas9-engineered *BLK*^*R131W/R131W*^ human B-cell line we demonstrated enhanced *IFNβ* expression in response to stimulation with the TLR7/8 agonist resiquimod (R848) (Fig. 4b).

We also asked whether the rare BLK variants found in SLE patients were more damaging than those found in healthy controls. For this we generated plasmids expressing each of the six rare BLK variants exclusively found in healthy controls (*BLK*^*P39L*^, *BLK*^*H55R*^, *BLK*^*K84N*^, *BLK*^*V101I*^, *BLK*^*R115Q*^, *BLK*^*G248A*^, Table 2) and tested side-by-side the repressive ability of all *BLK* variants. Five of six rare *BLK* variants found in patients with SLE had greater than 50% reduction in *IFNβ* repression (Fig. 4c) compared to wild-type BLK, whereas none of the six rare *BLK* variants found exclusively in healthy controls had a similar impairment (*p* < 0.0001). Consistent with the inability of SLE patient BLK variants to repress *IFNb*, examination of peripheral blood mononuclear cells from family G patients heterozygous for the *BLK*^*R238Q*^ variant (G.I.1, G.II.1, G.II.2, and G.II.3) revealed upregulation of the T1 IFN signature (Fig. 4c and module M1.2 in Fig. 4d) as well as apoptosis/survival pathways (module M6.6 in Fig. 4d, Supplementary Fig. 5). Together these data demonstrate that rare *BLK* variants in patients with SLE have impaired regulation of *IFNb* expression whereas those found only in healthy controls do not.

**Table 2:** Rare nonsynonymous *BLK* variants found in healthy controls

	MAF^a^	Type	AA change	CADD
*BLK*				
rs142352008	0.002	Missense	P39L	22.7
rs202053568	0.00003	Missense	H55R	5.8
rs778435147	0.00006	Missense	K84N	26.7
rs371256341	0.00002	Missense	V101I	5.2
rs367628135	0.00002	Missense	R115Q	25
rs763307492	0.000004	Missense	G248A	24.1

^a^Minor allelic frequency by gnomAD

### Variants in *Blk* augment pathogenic T cells in *Fas*^*lpr*^ mice

**Fig. 5:**
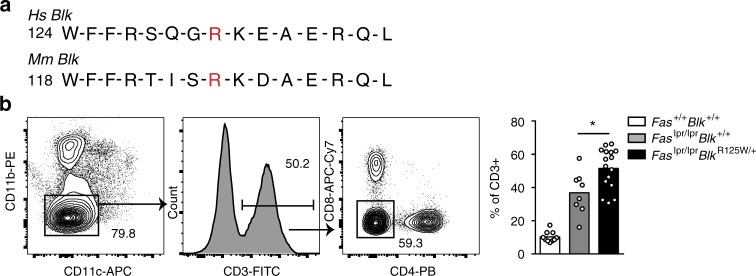
*Blk*^*R125W*^ exaggerates pathogenic double-negative T cells in *Fas*^*lpr/lpr*^ mice. **a** Sequence homology of human (Hs) BLK and murine (Mm) Blk around Hs *BLK*^*R131*^. **b** Representative contour plot of double-negative (DN) T cells from aged *Fas*^*lpr/lpr*^ mice crossed to the *Blk*^*R125W*^ mice (*p = 0.02, Mann–Whitney *U* test)

We next tested the effect of the *BLK* variants on lupus development in vivo using CRISPR/Cas9-generated mice bearing the orthologue of human *BLK*^*R131W*^, *Blk*^*R125W*^ (Fig. 5a). *Blk*^*R125W/R125W*^ mice had normal immune phenotypes comparable to those of heterozygous and wild type littermates (Supplementary Fig. 6A). Unlike the human *BLK*^*R131W*^ variant, stimulation of lymphocytes from CRISPR/Cas9-engineered mice expressing *Blk*^*R125W*^ did not reveal an increased T1 IFN response (Supplementary Fig. 6B) consistent with previous studies suggesting redundancy between *Blk* and other Src kinases in mice^37^. The *Blk*^*R125W*^ variant iwas introduced into mice with a genetic susceptibility to SLE. We chose C57BL/6.*Fas*^*lpr*^ mice because *Fas*.^*lpr*^ mice in the MRL genetic background develop a syndrome that resembles human lupus and *Blk* haploinsufficiency exacerbates lupus in MRL.*Fas*^*lpr*^ mice^26^. B6.*Fas*^*lpr*^ mice carrying a single *Blk*^*R125W*^ allele showed significantly expanded CD4/CD8 double-negative T cells (Fig. 5b), which have been shown to be important contributors to the hypercellularity in these mice and to provide help for autoantibody production in humans^38^. Together, these data suggest that human BLK phosphorylates IRF5 and the hypomorphic *BLK* alleles have impaired phosphorylation of IRF5 and diminished IRF5-mediated T1 IFN repression. Furthermore, a *BLK* allele orthologous to the one found in SLE was found to contribute to disease in lupus-prone mice.

### *BANK1* SNV enhances TRAF6-mediated nuclear IRF5 localization

**Fig. 6:**
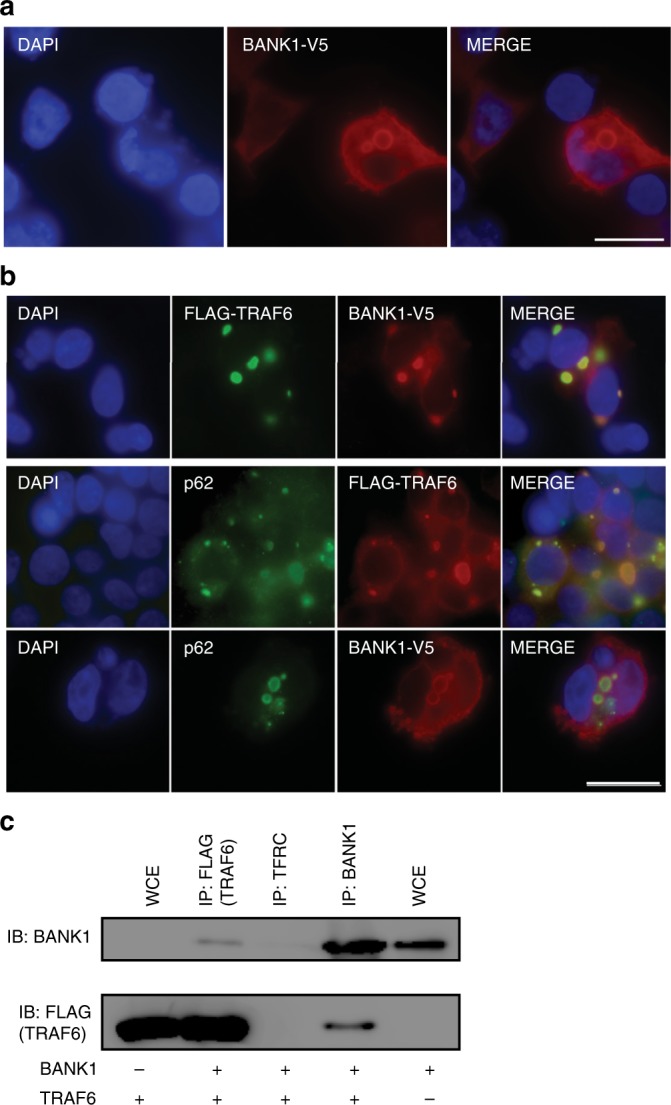
BANK^W40C^ impairs TRAF6 localization to p62 + sequestosomes. **a** Indirect immunofluorescence staining of HEK293T cells transfected with wild type BANK1-V5 and stained with a BANK1 antibody. Scale bar is 50 μm. DNA was stained with DAPI (blue). **b** Indirect immunofluorescence staining of HEK293T cells cotransfected with the cDNA constructs FLAG-TRAF6 and BANK1-V5 (top and middle panels) or with BANK1-V5 alone (bottom panel). Top panel: FLAG-TRAF6 was stained with a TRAF6 antibody (green) and BANK1-V5 was stained with a V5 antibody (red). Middle panel: FLAG-TRAF6 was stained with a TRAF6 antibody (red) and endogenous p62/SQSTM1 protein was labelled with a p62 antibody (green). Bottom panel: BANK1-V5 was stained with a BANK1 antibody (red) and endogenous p62/SQSTM1 protein was labelled with a p62 antibody (green). DNA was stained with DAPI (blue). Scale bar 50 μm. **c** Coimmunoprecipitation of BANK1-V5 and FLAG-TRAF6 from HEK293T cells demonstrating a two-way interaction. BANK1-V5 was pulled down using a BANK1 Ab and FLAG-TRAF6 with a FLAG antibody. TFRC transferrin receptor, isotype control, WCE whole-cytoplasmic extract

BANK1, initially identified as a substrate of LYN, is a scaffold protein lacking intrinsic kinase activity^39^. Scaffold proteins may positively and negatively regulate intracellular signalling pathways in innate and adaptive responses by controlling availability and post-translational modification of signaling proteins^40^. When expressed in HEK293T cells BANK1 formed cytoplasmic inclusion bodies in a proportion of cells (Fig. 6a) reminiscent of TRAF6-containing sequestosomes. Indeed, we confirmed wild type BANK1 colocalized with TRAF6, the sequestosome protein p62 (Fig. 6b), as well as with the deubiquitinating enzyme CYLD, which plays a critical role in regulating TLR signaling by deubiquitinating TRAF6^41^ (Supplementary Fig. 7A). BANK1 also localized in cytoplasmic aggregates when co-expressed with MYD88 (Supplementary Fig. 7B). We confirmed that BANK1 formed a complex with TRAF6 by coimmunoprecipitation upon expression in HEK293T cells (Fig. 6c).

**Fig. 7:**
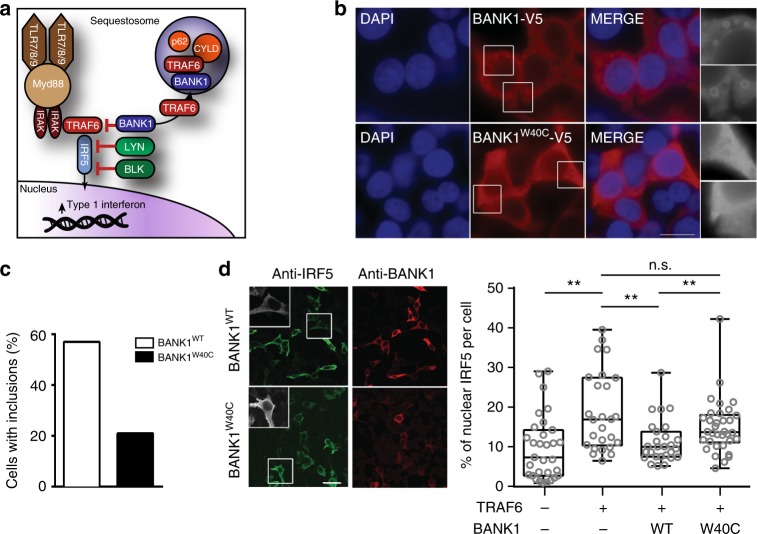
Impaired sequestration of TRAF6 by BANK1 increases nuclear IRF5. **a** Schematic of the role of *BANK1* and *BLK* on regulation of T1 IFN production. Red lines indicate negative regulation. **b** Indirect immunofluorescence staining of HEK293T cells expressing either wild type (top panels) or W40C (bottom panels) BANK1-V5 cDNA constructs. BANK1 protein was detected using an antibody specific for the V5 tag (red). DNA was stained with DAPI (blue). Scale bar 20 μm. **c** Quantification of the percentage of HEK293T cells expressing WT or W40C BANK1-V5 with cytoplasmic inclusion bodies. Cells were transfected with 3 μg of the BANK1-V5 cDNA constructs and stained 72 h post transfection. Greyscale inserts show enlargements of the indicated cell sections. Double blind cell counting was carried out on at least 100 cells per experiment with three experimental replicates. **d** Representative images of indirect immunofluorescence staining of HEK293T cells expressing IRF5, FLAG-TRAF6 and either WT BANK1-V5 (top panel) or W40C BANK1-V5 (bottom panel). IRF5 protein was detected using an antibody specific for IRF5 (green) and BANK1 was detected using an antibody recognizing the V5 tag (red). Greyscale inserts show enlargements of the indicated cell. Scale bar 50 μm. Double blind cell counting was carried out on at least 100 cells per experiment with *n* = 3 experimental replicates. Bar graph represents quantification of the percentage of nuclear IRF5 as a proportion of the total IRF5 per cell based on indirect immunofluorescence staining of HEK293T cells expressing IRF5, FLAG-TRAF6 and either WT BANK1-V5 or W40C BANK1-V5 (as in (**d**)). Quantification was performed using the ImageJ software^64^. (Centre line = median, box = 25th to 75th percentile, whiskers = range; *p < 0.05, **p < 0.01, Mann–Whitney *U*, representative of two experimental replicates)

TRAF6 is known to ubiquitylate IRF5 resulting in activation and subsequent nuclear localization of IRF5 and T1 IFN production^42^. We thus hypothesized that BANK1 promotes sequestration of TRAF6 in typical p62^+^ and CYLD^+^ sequestosomes, reducing TRAF6 ubiquitination and thereby dampening IRF5 activation and induction of TI IFN (Fig. 7a). Consistent with this hypothesis, expression of *BANK1*^*W40C*^ lead to significantly reduced formation of BANK1^+^ sequestosomes (Fig. 7b, c). Furthermore, when expressed with *TRAF6* and *IRF5*, WT BANK1 significantly repressed TRAF6-mediated IRF5 nuclear localization. By contrast, *BANK1*^*W40C*^ could not repress TRAF6-mediated IRF5 nuclear localization to the extent of WT BANK1 (Fig. 7d). These findings demonstrate that the scaffold protein BANK1 regulates TRAF6 activity and hence IRF5 signaling, and establish that *BANK1*^W40C^ is a loss-of-function variant that promotes T1 IFN activity.

## Discussion

Although SLE is the result of a combination of environmental and intrinsic predispositions, genetic risk remains one of the most potent risk factors^9^. GWAS have provided substantial advances in identifying possible disease pathways, yet they have been less informative about disease mechanisms^43^. This is because variants identified by GWAS, which are typically found at high frequencies, only modestly increase risk and in the significant majority of cases have modest or no effect on protein function^43^. The prevalent hypothesis to explain substantial genetic risk for SLE with common, weak variants is that risk arises from the cumulative burden of dozens of these GWAS alleles. However, the expanding, but still small, list of monogenic causes of SLE supports the notion that novel or rare gene variants that significantly cripple the function of crucial DNA-sensing or degrading enzymes, or complement factors involved in the removal of apoptotic cells, can cause severe and often early onset SLE-like disease^20^. We show here that SLE patients are likely to harbour two or more rare variants in genes implicated in SLE by GWAS or involved in the regulation of T1 IFN. The main focus of the study was to determine whether the variants found in SLE patients were damaging and could contribute to disease, and if so, whether they were more damaging than those found in controls.

We tested the functional consequences of mutations in two genes: *BLK*, which harboured novel or rare variants in 10.5% of SLE probands, and *BANK1*, encoding a known BLK interacting partner and shown to act in epistasis with *BLK*^30^. Significantly, we demonstrate that these rare variants exert measurable damaging effects on protein function, ultimately leading to a common endpoint of increased T1 IFN activity in human B cells. Excessive TI IFN activity is a unifying feature in the majority of SLE patients^44^. Moreover, since *BANK1* and *BLK* are also expressed in plasmacytoid dendritic cells^26,28,29^, it is likely that a similar failure to repress TI IFN occurs in these cells, which are major producers of these cytokines^45^. Thus, these data indicate that rare SNVs in the GWAS-implicated genes *BANK1* and *BLK* are associated with development of lupus and related autoimmune diseases.

We identify a novel role for BLK in regulating T1 IFN downstream of TLR7/8 signalling, and demonstrate that the rare variants found in SLE patients impair the ability of BLK to repress T1 IFN production. The incomplete penetrance of autoimmunity in *BLK* heterozygous individuals and the absence of overt autoimmunity in *Blk*^*R125W/R125W*^ mice may be due to the differences in environmental exposure to stimulants of T1 IFN such as viral infection. It is also likely that there is increased redundancy of Blk in mice, in which other Src family-kinases have been shown to compensate for Blk deficiency^37^. Moreover, the presence of additional lupus-predisposing gene variants in SLE patients may enhance the BLK defects. Strikingly, we observed a distinct difference in the deleteriousness of rare variants in *BLK* in HC and SLE, suggesting that quality (degree of damage to protein function) rather than quantity (number of rare variants) may be a more important determinant of contribution of rare variants to disease.

BANK1, a scaffold protein, lacks intrinsic kinase activity and may regulate signalling events by localization or sequestration of significant intracellular signalling proteins. Here, we show that a low-frequency (MAF < 2%) *BANK1* variant diminishes localization of BANK1 to sequestosomes, likely altering recruitment of TRAF6 and CYLD to these regulatory structures. This is associated with enhanced nuclear localization of IRF5, which is a positive regulator of TI IFN transcription^46^. Since BANK1 is a direct target of BLK phosphorylation, and BANK and BLK have been previously shown to act in epistasis^30^, BLK’s capacity to repress TI-IFN is probably related to its ability to regulate BANK1 localization to sequestosomes and as a consequence, regulate sequestosome homeostasis and TRAF6 activity.

Our demonstration that rare gene variants in GWAS-identified SLE risk genes are damaging, and that these occur in a large fraction of SLE patients supports the notion that rare variants, as shown in other common and genetically complex conditions^16,17^, contribute to SLE pathogenesis. Furthermore, the co-segregation of variants in *BANK1* and *BLK* in two families suggests that sporadic lupus may occur upon inheritance or de novo occurrence of two or more rare variants with strong effects that act together to cause disease.

Together, our findings demonstrate a role of rare *BLK* and *BANK1* variants in SLE and may offer an alternative explanation for the association of some common variants in linkage disequilibrium. Identification of rare variants as causes of lupus and related systemic autoimmune disorders through whole exome sequencing provides an approach intermediate between GWAS and conventional mapping. An approach that demands functional verification, but if provided, as we show here, can also yield novel insights into disease mechanisms and novel targets for treatment.

## Methods

### Human patients and DNA sequencing

Written informed consent was obtained as part of the Australian Point Mutation in Systemic Lupus Erythematosus study (APOSLE) and the Centre for Personalised Immunology program. The study was approved by and complies with all relevant ethical regulations of the Australian National University and ACT Health Human Ethics Committees. SLE cohort 1 and 2 were both recruited in Australia, processed and sequenced on similar platforms. These two cohorts were recruited sequentially, with SLE cohort 1 recruited between 2008 and 2014 and SLE cohort 2 recruited between 2015 and 2017. Saliva was collected in Oragene™ DNA self-collection kits and purified using PrepIT™ DNA purification kits (Oragene) and treated with Ribonuclease A (Qiagen Cat# 19101). DNA samples were enriched with Human SureSelect XT2 All Exon V4 Kit and sequenced by Illumina HiSeq 2000 (Illumina, Inc.). WES had 21% low or uncovered exon bases compared with 4% low or uncovered exon bases for WGS. Bioinformatic analysis was performed at JCSMR, ANU. Raw sequence reads were aligned to the reference genome (Hg19) and single-nucleotide variants and small insertions and deletions called using GATK. Results were scored based on reported minor allelic frequency (MAF), Polyphen2 score, expression in immune tissues and reported mouse phenotypes. All SNVs of interest in *BLK* and *BANK1* were confirmed by Sanger sequencing. Amplifluor to detect *BANK1*^*W40C*^ and *BLK*^*R131W*^ in the APOSLE cohort was performed using the CHEMICON Amplifluor SNPs HT Genotyping System Fam-Joe kit S7909 (Merck-Millipore). The 45 and Up^47,48^ and ASPREE^49^ datasets were used as reference healthy controls, accessed through the MGRB Collaborative (http://sgc.garvan.org.au/mgrb/initiatives).

### WES/WGS data processing and batch correction

Probes were filtered out if the detection *p* value was greater than 0.01 for at least 100% of the samples. All data values <10 were set to 10 and then the data was log2 transformed. An additional filter selecting the 75% most variable transcripts was performed, leaving a total 18,004 probes for analysis. Principal variance component analysis (PVCA) was conducted to identify undesirable sources of technical variability within the data and batch correction was applied to correct for this technical variation. Both PVCA and batch correction were conducted using JMP Genomics 7.0 (SAS Institute) analysis software.

### Determination of ethnicity by WES/WGS

We determined each individual’s ethnicity utilizing GEMTools^50–52^ with genotypes across 23,556 sites^53^ extracted from all 2504 Phase 3 samples of the 1000Genomes project^54^ to cluster our 230 samples individually into corresponding 1000Genomes superpopulations which directly correspond to 6 populations within gnomAD (AFR, AMR, EAS, FIN, NFE, and SAS). In all 230 GEMTools clustering runs (where the only nondefault parameter was setting maximum individual cluster size to 114 to match the maximal sample size of 1000Genomes’ 26 populations), each of our samples fell unambiguously within a population-homogenous cluster which was assigned as its best-matching population. Admixture was determined in our sample cohorts using rADMIXTURE, an implementation of the ADMIXTURE algorithm^55^ to correct for population stratification, applied on the Dodecad K7b (http://dodecad.blogspot.com/2012/01/k12b-and-k7b-calculators.html) global ancestry reference panel. This method, though accurate for determining ancestral population components across continents, was not suited for binning our samples into an admixed gnomAD population like AMR, thus we used our GEMTools-derived best-matching population to select each sample’s ethnically matched gnomAD population frequency.

### RNA-expression analysis

Whole blood was collected in acid citrate dextrose (ACD) tubes. RNA was extracted from whole blood (5′ Prime Perfect Pure kit) and stored at −80 °C until use. Differential gene expression analysis was performed using linear modeling with the Limma package^56^. Gene-set analysis was conducted using the QuSAGE algorithm^57^, which tests whether the average log2-fold change of a gene set is different from 0 and takes into account the correlations of the genes by incorporating an estimate of the variance inflation factor of the gene set. Module maps were generated as reported previously^58^.

### Expression vectors and mutagenesis

The following expression vectors were obtained: untagged BLK (OriGene Technologies), myc-LYN (GeneCopoeia, Inc.), untagged IRF5 (OriGene Technologies). BANK1-V5 was a gift from C. Castillejo-López (Uppsala University). Mutagenesis was performed using the Quikchange I and II Site directed mutagenesis protocols (Agilent Technologies). IFNβ luciferase (Addgene)^59^ pRL-CMV (Promega). pCMV-HA-MyD88 was a gift from Bruce Beutler (Addgene plasmid # 12287), pX330-U6-Chimeric_BB-CBh-hSpCas9 was a gift from Feng Zhang (Addgene plasmid # 42230).

### Antibodies

Antibodies for western blotting, immunofluorescence imaging, and coimmunoprecipitation studies were as follows: mouse anti-human BLK (SC-65980; Santa Cruz), Mouse anti-V5 (clone SV5-Pk1, MCA1360, BioRad); mouse anti-FLAG M2 (F1804; Sigma); Rabbit anti-LYN (06–207, EMD Millipore); mouse anti phospho tyrosine-horseradish peroxidase (HRP) (R&D Systems); IRF5 (Abcam) and monoclonal mouse anti-CD71 (transferrin receptor) antibody (C2063, Sigma). Secondary antibodies were conjugated to HRP (Jackson ImmunoResearch), Alexa 568 or Alexa 488 (Molecular Probes, Invitrogen). Indo-1 AM was from ThermoFisher and anti-IgM from Jackson Immunoresearch. All FACS and microscopy work was carried out at the Microscopy and Cytometry Facility, Australian National University.

### Flow cytometry

The study was approved by and mouse handling complies with all relevant ethical regulations of the Australian National University Ethics Comittee. Spleens were isolated as single-cell suspensions after red blood cell lysis. To stain for surface markers, we incubated cells in the antibody mixture diluted in ice-cold staining buffer (2% fetal calf serum in phosphate-buffered saline). Ramos cells were loaded with Indo-1 AM at 37 °C for 2 h before being stimulated at 37° with anti-Fab(2) antibody. An LSRII or Fortessa Flow Cytometer with FACSDiva software were used for flow cytometry acquisition, and FlowJo (Tree Star) was used for analysis.

### Transfection, immunoprecipitation, and western blotting

HEK 293T cells were transfected (Lipofectamine 2000; Life Technologies) with the relevant plasmids as per manufacturer’s recommendation. Cells were lyzed using NP-40 lysis buffer and immunoprecipitated with the relevant antibody using Protein G Sepharose (GE Healthcare) and the relevant antibody. For coimmunoprecipitation experiments transferrin receptor was used as isotype control. Immunoprecipitants were resuspended in SDS (sodium dodecyl sulfate)-buffer and boiled prior to electrophoresis on 8% SDS-polyacrylamide gel electrophoresis gels. Gels were transferred to nitrocellulose membranes (BioRad Laboratories), blocked overnight (TBST + skim milk powder; or 5% bovine serum albumin for phosphotyrosine blots) and probed with the relevant primary and secondary antibodies. Membranes were developed with enhanced chemiluminescence developer (Western Lightning Plus ECL; Perkin Elmer).

### DLAs and electroporation

HEK293T cells were transfected with an IFN-β luciferase reporter, pRL-CMV (10 ng; Promega) Renilla luciferase control reporter, pcDNA 3.1 and indicated vectors and 24 h later dual luciferase assays (DLAs) were performed as per published protocols^60,61^. For CpG response assays, TLR9-HEK239s (Invivogen) were transfected using lipofectamine with indicated vectors, IFNβ-luciferase and renilla reporters before being stimulated after 24 h with 5 µg/ml CpG (ODN 2006, InvivoGen). The Burkitt’s lymphoma cell line Ramos was nucleofected with the IFN-β luciferase reporter and pRL-CMV using the NEON transfection system (ThermoFisher Scientific) according to the manufacturer’s instructions.

### CRISPR-Cas9 genome editing of human B-cell lines

The vector px330^62^ (Addgene), which expresses Cas9 and the sgRNA, was linearized with *Bbs*I and gel-purified. A pair of complementary 18mer oligos targeting a single site within the genome (primer sequences available upon request) were annealed and ligated to the linearized vector. pX330 expressing the sgRNA of interest was transfected along with an mcherry plasmid into the Burkitt’s lymphoma cell line Ramos using the NEON transfection system (ThermoFisher Scientific) according to the manufacturers’ instructions. mcherry^+^ single cells were sorted 24–48 h later into 96-well plates and individual clones expanded until confluent. gDNA was extracted by digesting cells with proteinase K (0.1% Tween20, 100 μM EDTA, 500 μg/mL Proteinase K, 1× high fidelity Phusion buffer) at 56 °C for 40 min, followed by incubation at 95 °C for 8 min. A region of approximately 300 bp flanking the variant was PCR amplified (primer sequences available on request) using Phusion DNA Polymerase II (ThermoFisher Scientific) and presence of the mutation confirmed by Sanger sequencing. All sequencing was carried out at the ACRF *Biomolecular Resource Facility* and Genome Discovery Unit, Australian National University.

### CRISPR-Cas9-mediated genome-editing of mouse zygotes

C57BL/6 mice were housed under specific pathogen-free conditions. All mouse procedures have been approved by the Australian National University Animal Experimentation Ethics Committee. (AEEC A2014/058 and A2014/016) under the NHMRC Australian code of practice. *Blk* gRNA and Cas9 protein were obtained from PNABio. Oligo and ssOligos were purchased from IDT (sequences available on request). C57BL/6Ncrl female mice (4–5 weeks old) were superovulated with Pregnant Mare Serum Gonadotrophin (PMSG) 5UI day 1 and Human Chorionic Gonadotrophin hormone (HCG) 5UI day 3. After detection of a vaginal plug of the superovulated females, mouse zygotes were harvested from the ampullae and were placed in KSOM medium (Sigma). Cas9n protein (100 ng/µl) was co-injected with a mixture of sgRNA (50 ng/µl each) and ssOligo (100 ng/µl) into the cytoplasm of the fertilized eggs into M2 medium (Sigma). After micro-injection, the zygotes were incubated overnight at 37 °C and 5% CO_2_ and two-cell stage embryos were surgically transferred into the uterus of pseudopregnant CD1 recipient females at 2.5 dpc. Three weeks after birth mouse ears were punched. DNA was extracted and Sanger sequencing performed to confirm the mutations. All the mouse zygote preparation and micro-injection was carried out at the Australian Phenomics Facility, Australian National University. The sequencing was carried out at the ACRF *Biomolecular Resource Facility* and Genome Discovery Unit, Australian National University.

### qPCR

Total RNA was extracted from splenocytes stimulated in the presence of R848 for 24 h using Trizol (Invitrogen). cDNA was synthesized using superscript III (Thermo) or miscript (Qiagen) according to manufacturer’s instructions. Quantitative PCR (qPCR) was carried out using the SYBR green method with the following primer sequences; *Eif2ak* (forward, 5′-ATGCACGGAGTAGCCATTACG-3′; reverse-GACAATCCACCTTGTTTTCGT-3′)*, If44* (forward, 5′-AACTGACTGCTCGCAATAATG-3′; reverse- GTAACACAGCAATGCCTCTTGT-3′)*, Ifih1* (forward, 5′-AGATCAACACCTGTGGTAACACC-3′; reverse-CTCTAGGGCCTCCACGAACA-3′)*, Igs15* (forward, 5′- GGTGTCCGTGACTAACTCCAT-3′; reverse-TGGAAAGGGTAAGACCGTCCT-3′)*, Irf7* (forward, 5′-GAGACTGGCTATTGGGGGAG-3′; reverse-GACCGAAATGCTTCCAGGG-3′)*, Oas2* (forward, 5′-AGTTCCTACTGACCCAGATCC-3′; reverse- AGAGGGCTCTTACTGGCACTT-3′)*, Oas3* (forward, 5′-TCTGGGGTCGCTAAACATCAC-3′; reverse- GATGACGAGTTCGACATCGGT-3′)*. Ct* values were normalized to *Gapdh* (forward, 5′-AATGTGTCCGTCGTGGAT-3′; reverse-CTCAGATGCCTGCTTCAC-3′) and relative expression was calculated using the 2^−^^ΔΔ*C*t^ method^63^.

## Supplementary information

Supplementary Information

Description of Additional Supplementary Files

Supplementary Data 1

Supplementary Data 2

## Data Availability

WES and WGS sequencing data of the 76 genes described in this article have been deposited to the EGA database under the accession number EGAD00001004859 and are accessible to all qualified researchers meeting the data access policy (EGAP00001001134) as determined by the Data Access Committee (EGAC00001001157). The 45 and Up^47,48^ and ASPREE^49^ datasets were used as reference healthy controls, accessed through the MGRB Collaborative (http://sgc.garvan.org.au/mgrb/initiatives) and are accessible to all qualified researches meeting the MGRB data access policy (https://sgc.garvan.org.au/terms/mgrb). All variants have been submitted to ClinVar (SUB4880889 and SUB5484273). The microarray data have been deposited to the GEO database under the accession number GSE126307.
