# Supplementary material for: Functional rare and low frequency variants in BLK and BANK1 contribute to human lupus
Source: Nat Commun. 2019 May 17;10:2201. doi: 10.1038/s41467-019-10242-9 (PMC6525203; doi:10.1038/s41467-019-10242-9)
Supplement: Supplementary file 1 — Supplementary Information [file 41467_2019_10242_MOESM1_ESM.pdf]

Functional rare and low frequency variants in *BLK* and *BANK1* contribute to human lupus pathogenesis

Jiang et al.

Supplementary Figure 1

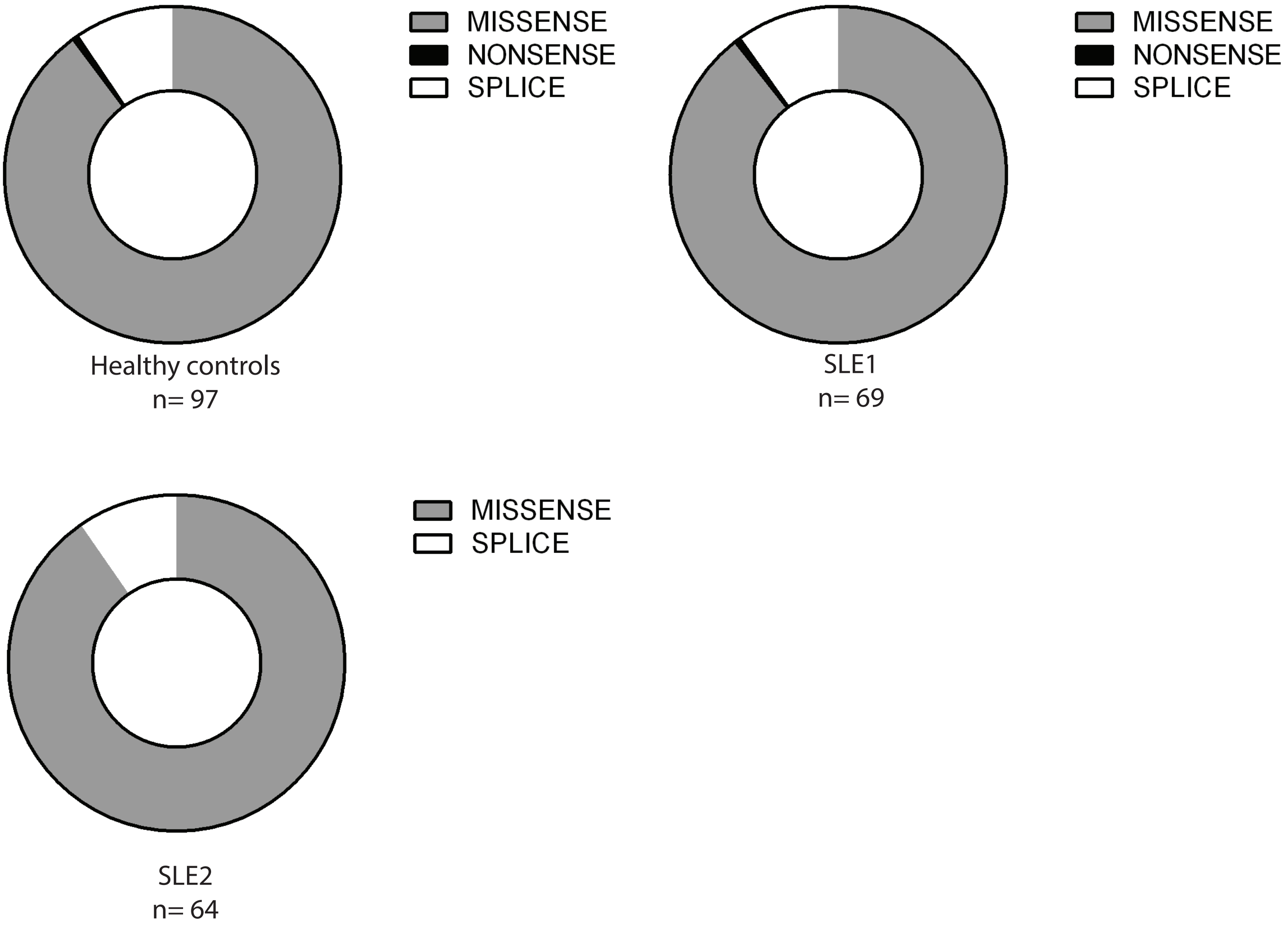

Supplementary Figure 1
Figures representing frequency of missense, nonsense, and splice variants in health controls, SLE1 and SLE2 cohorts.

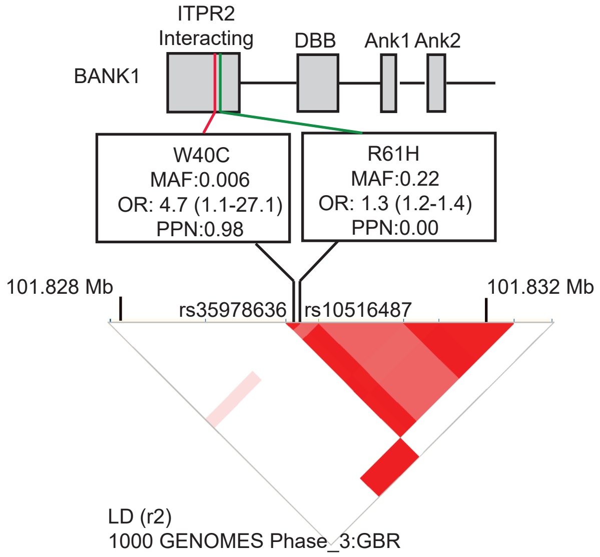

Supplementary Figure 2

Linkage disequilibrium LD (r2) plot (adapted from Ensembl) between the rare BANK1W40C variant and the common GWAS-associated SLE risk allele BANK1R61H in the 1000GENOMES:phase_3:GBR population (British in England and Scotland), with schematic of SNV positions within IP3R-2 protein domain. DBB (Dof, BCAP, BANK motif), ITPR2 (inositol 1, 4, 5-triphosphate receptor type 2), ANK (ankyrin).

.

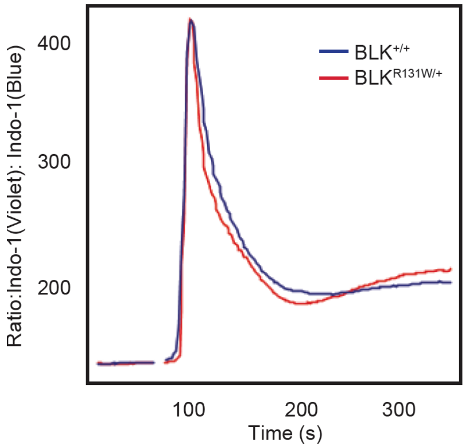

Supplementary Figure 3
Ca2+ flux of CRISPR-Cas9 edited Ramos cells.

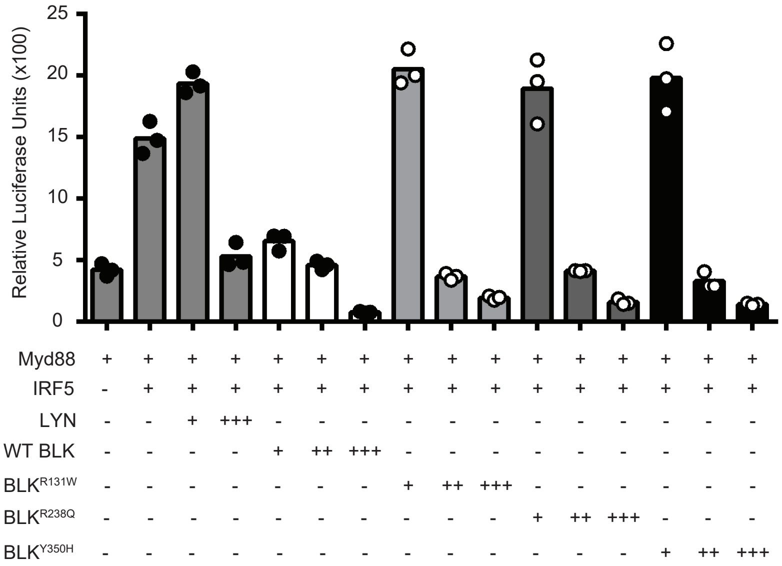

Supplementary Figure 4
IFNβ luciferase activity 24hrs after co-transfection of HEK293T with IRF5, MyD88, LYN and indicated BLK variants. Amount of DNA transfected +=50ng; ++=100ng; +++200ng.

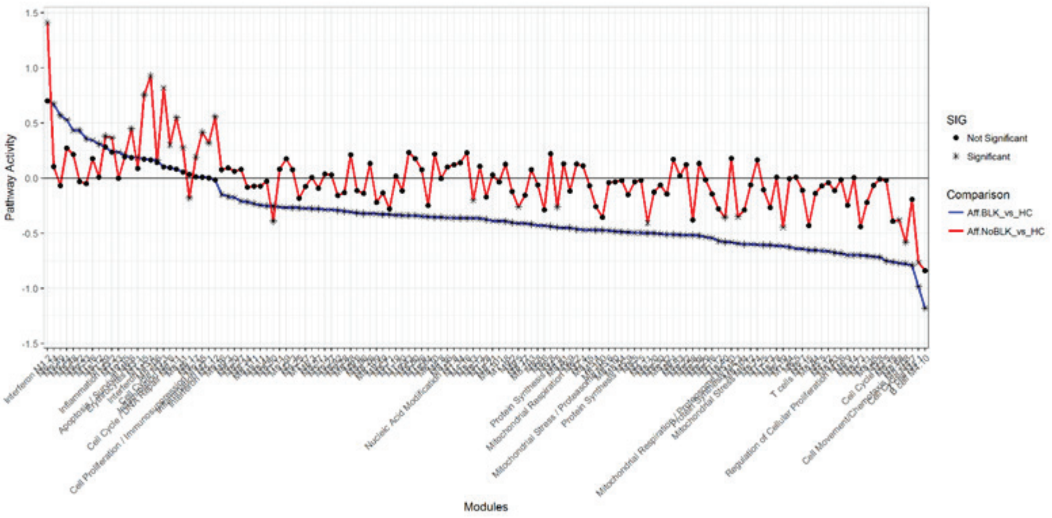

Supplementary Figure 5
The module level log2 fold change estimates from QuSAGE for the BLK vs healthy and non BLK vs healthy comparisons. The modules plotted are significant (FDR <= .05) in at least one of the comparisons and ordered from greatest positive change to greatest negative change in the BLK vs healthy comparison. IFN module M1.2 is furthest to the left and is the most upregulated for both BLK and non BLK patients.

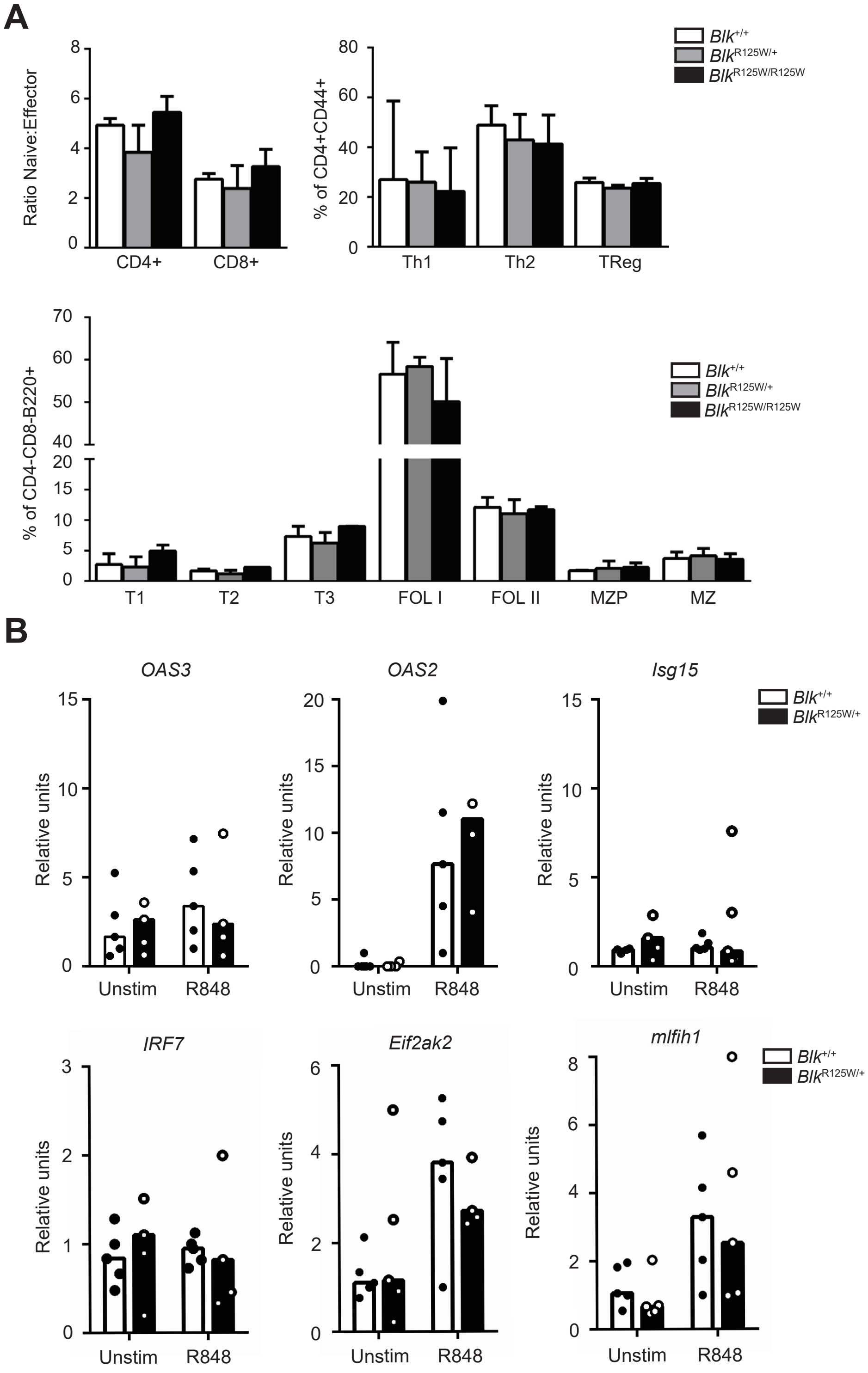

Supplementary Figure 6

A) Immunophenotyping of 12 week-old mice of indicated genotype. B) qPCR of IFN-responsive genes in total splenocytes from BLKR125W/+ mice 24hrs after stimulation with resiquimod (R848).

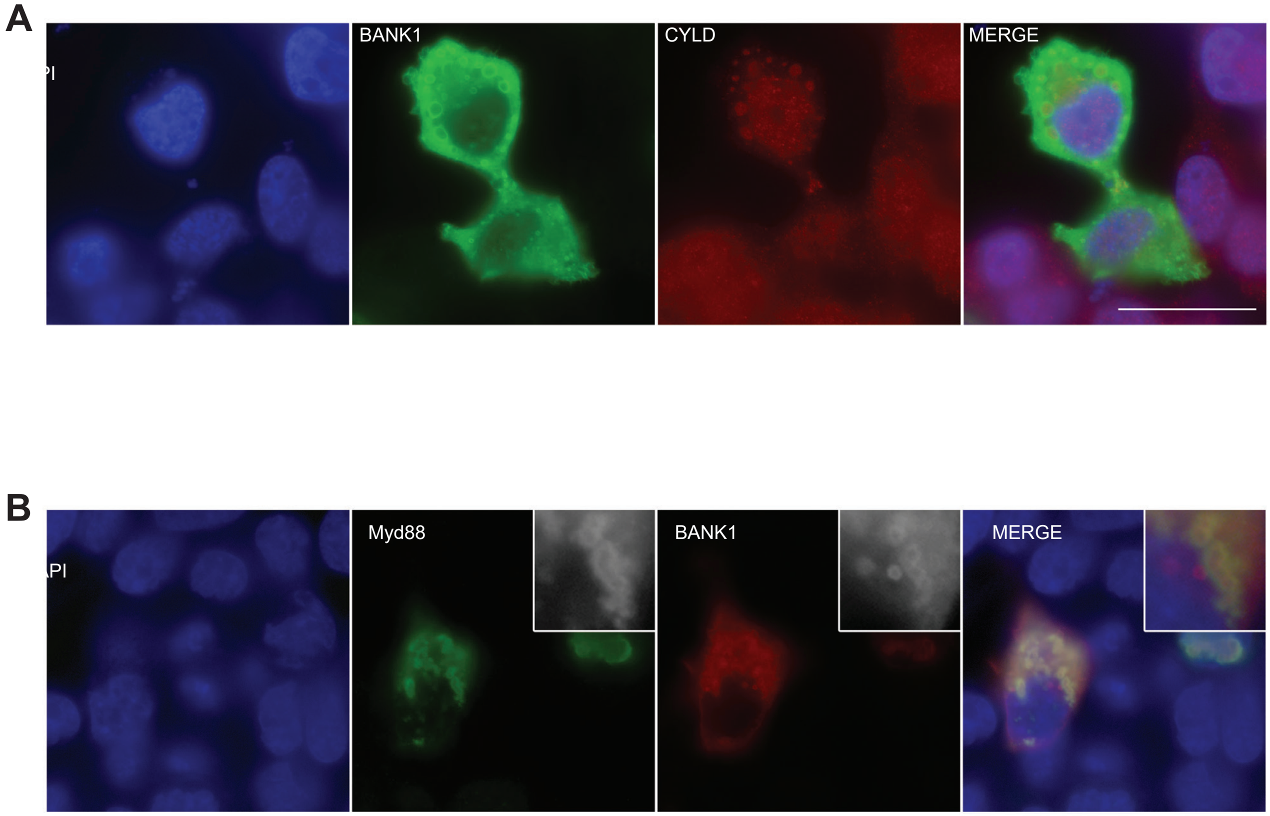

Supplementary Figure 7

A) Indirect immunofluorescence staining of HEK293T cells transfected with wild type (WT) BANK1-V5 and stained with anti-BANK1 and anti-CYLD. B) Indirect immunofluorescence staining of HEK293T cells transfected with wild type (WT) BANK1-V5 and stained with anti-BANK1 and anti-MyD88. Scale bar 50 µm.

Supplementary Figure 8

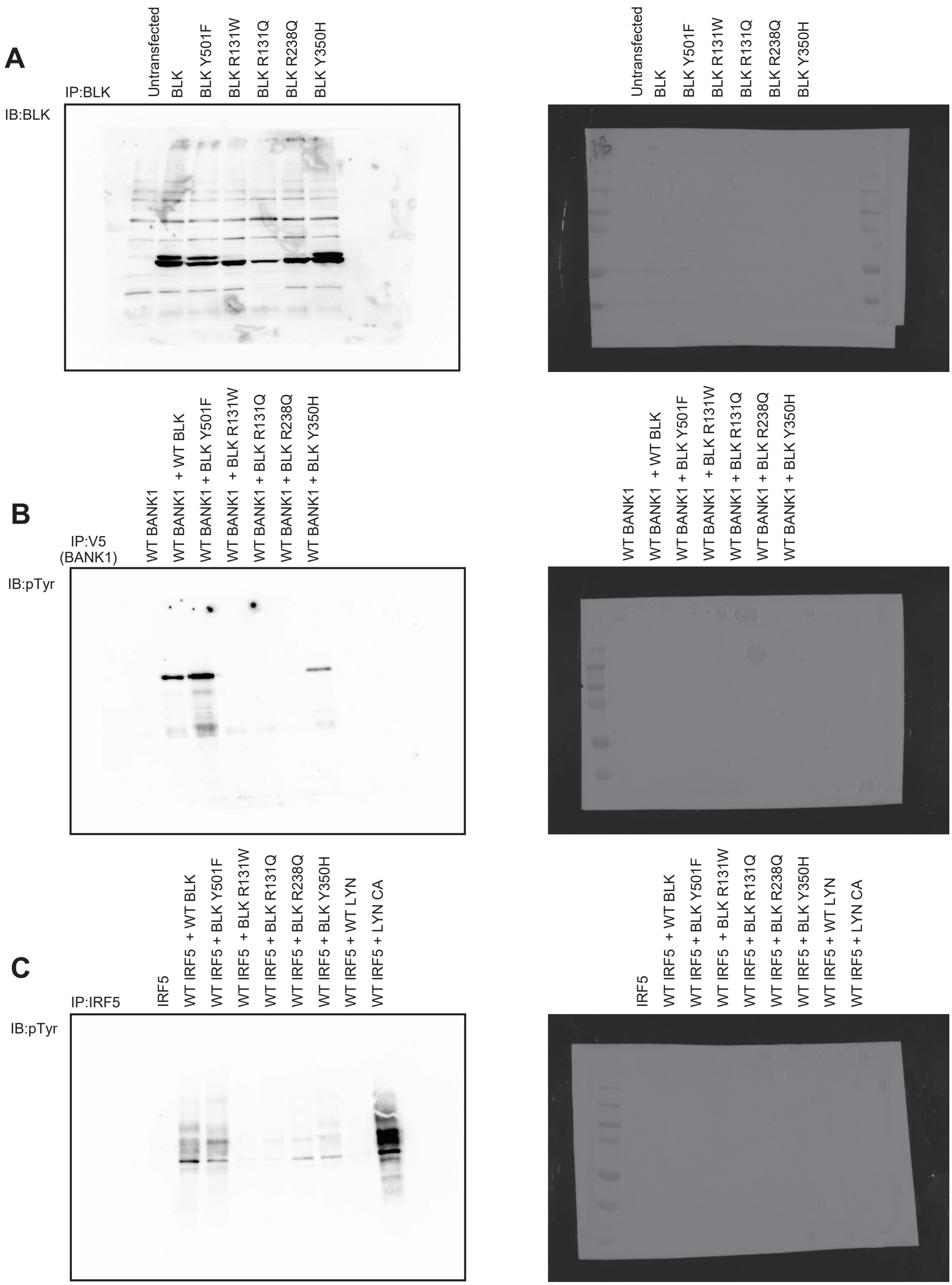

Supplementary Figure 8

Uncropped blots and corresponding digital image of protein ladder for A) Fig. 3B, B) Fig. 3C, C) Fig. 3D
