# Supplementary material for: Functional rare and low frequency variants in BLK and BANK1 contribute to human lupus
Source: Nat Commun. 2019 May 17;10:2201. doi: 10.1038/s41467-019-10242-9 (PMC6525203; doi:10.1038/s41467-019-10242-9)
Supplement: Supplementary file 2 — Description of Additional Supplementary Files [file 41467_2019_10242_MOESM2_ESM.pdf]

Description of Additional Supplementary Files

File Name: Supplementary Data 1
Description: Characteristics of SLE cohort describing ethnicity, age of diagnosis and method of sequencing.

File Name: Supplementary Data 2
Description: List of 76 SLE and T1 IFN associated genes and the frequency of their rare variants by cohort.
